# The vaginal microbiome of pregnant women is less rich and diverse, with lower prevalence of Mollicutes, compared to non-pregnant women

**DOI:** 10.1038/s41598-017-07790-9

**Published:** 2017-08-23

**Authors:** Aline C. Freitas, Bonnie Chaban, Alan Bocking, Maria Rocco, Siwen Yang, Janet E. Hill, Deborah M. Money, The VOGUE Research Group, Sean Hemmingsen, Gregor Reid, Tim Dumonceaux, Gregory Gloor, Matthew Links, Kieran O’Doherty, Patrick Tang, Julianne van Schalkwyk, Mark Yudin

**Affiliations:** 1 Department of Veterinary Microbiology, University of Saskatchewan, Saskatoon, SK S7N 5B4 Canada; 2 Departments of Obstetrics and Gynecology and Physiology, University of Toronto, Toronto, ON M5G 1L4 Canada; 3 Lunenfeld-Tanenbaum Research Institute, Toronto, Ontario M5T1×5 Canada; 4 Department of Obstetrics and Gynaecology, University of British Columbia, Vancouver, BC V6T 1Z4 Canada; 5 Women’s Health Research Institute, BC Women’s Hospital & Health Centre, Vancouver, BC V6H 3N1 Canada; 6 Faculty of Science, Health, Education and Engineering, Present Address: University of the Sunshine Coast, Queensland, Australia; 7 National Research Council Canada, Saskatoon, SK Canada; 8 Department of Microbiology & Immunology, University of Saskatchewan, Saskatoon, SK Canada; 9 Department of Microbiology and Immunology, University of Western Ontario, London, ON Canada; 10 Lawson Health Research Institute, London, ON Canada; 11 Saskatoon Research and Development Centre, Agriculture and Agri-Food Canada, Saskatoon, SK Canada; 12 Department of Biochemistry, University of Western Ontario, London, ON Canada; 13 Department of Animal & Poultry Science, University of Saskatchewan, Saskatoon, SK Canada; 14 Department of Psychology, University of Guelph, Guelph, ON Canada; 15 Department of Pathology, Sidra Medical and Research Center, Doha, Qatar; 16 Department of Obstetrics & Gynaecology, University of Toronto, Toronto, ON Canada; 17 Department of Obstetrics and Gynecology, St. Michael’s Hospital, Toronto, ON Canada

## Abstract

The vaginal microbiome plays an important role in maternal and neonatal health. Imbalances in this microbiota (dysbiosis) during pregnancy are associated with negative reproductive outcomes, such as pregnancy loss and preterm birth, but the underlying mechanisms remain poorly understood. Consequently a comprehensive understanding of the baseline microbiome in healthy pregnancy is needed. We characterized the vaginal microbiomes of healthy pregnant women at 11–16 weeks of gestational age (n = 182) and compared them to those of non-pregnant women (n = 310). Profiles were created by pyrosequencing of the *cpn*60 universal target region. Microbiome profiles of pregnant women clustered into six Community State Types: I, II, III, IVC, IVD and V. Overall microbiome profiles could not be distinguished based on pregnancy status. However, the vaginal microbiomes of women with healthy ongoing pregnancies had lower richness and diversity, lower prevalence of *Mycoplasma* and *Ureaplasma* and higher bacterial load when compared to non-pregnant women. *Lactobacillus* abundance was also greater in the microbiomes of pregnant women with *Lactobacillus*-dominated CSTs in comparison with non-pregnant women. This study provides further information regarding characteristics of the vaginal microbiome of low-risk pregnant women, providing a baseline for forthcoming studies investigating the diagnostic potential of the microbiome for prediction of adverse pregnancy outcomes.

## Introduction

The complex microbial community present in the female lower genital tract is an important factor in a woman’s reproductive health. Imbalances in this microbiota can lead to bacterial vaginosis (BV), one of the most common gynaecologic conditions in women of reproductive age throughout the world^1–3^. At present, it is understood that the healthy vaginal microbiome is dominated by *Lactobacillus* species, while BV is characterized by a relatively low abundance of *Lactobacillus* spp. accompanied by polymicrobial anaerobic overgrowth, including species such as *Gardnerella vaginalis*, *Prevotella* spp., *Bacteroides* spp., *Mobiluncus* spp., and *Mycoplasma hominis* ^4, 5^.

In pregnancy, such imbalances in the vaginal microbiome are associated with an increased risk of post-abortal infection^6, 7^, early^8, 9^ and late miscarriage^10, 11^, histological chorioamnionitis^12, 13^, postpartum endometritis^14, 15^, preterm premature rupture of membranes (PPROM)^16, 17^ and preterm birth^18^. The relationship between BV and preterm birth in particular has profound implications since children who are born prematurely have higher rates of cardiovascular disorders, respiratory distress syndrome, neurodevelopmental disabilities and learning difficulties compared with children born at term^19^, along with increased risk of chronic disease in adulthood^20^. Also, preterm birth complications are estimated to be responsible for 35% of the world’s annual neonatal deaths^21^.

The development of culture-independent techniques, such as high throughput DNA sequencing, has greatly facilitated the comprehension of the composition and role of the vaginal microbial community. These tools can be used to exploit the potential of the microbiome to diagnose BV-associated conditions. However, while most studies have focused on the vaginal microbiome of healthy, non-pregnant, reproductive aged women, relatively little is known about this microbiome in pregnancy. Pregnancy is associated with a variety of physiological events including increased sex steroid hormone levels^22^, host immune response modulation^23, 24^, altered immune-physicochemical properties of the cervical mucus^25–27^, as well as behavioural changes such as reduced drinking and smoking^28^. These factors may drive changes in the structure and/or composition of the microbial community resulting in a microbiome that is different from that of non-pregnant women. Thus, the current definition of a healthy microbiome, which has been based on findings in non-pregnant women, cannot necessarily be extrapolated to the microbiome in pregnancy. Establishing baseline data for the vaginal microbiome during pregnancy is a crucial step in the process of developing tools to predict and prevent pregnancy complications such as preterm birth, which is in turn associated with infection and/or inflammation as well as significant infant mortality and morbidity.

A few recent studies have used an amplicon sequencing approach to analyze the vaginal microbiome in pregnancy, all of which have been based on amplification and sequencing of variable regions of the 16 S rRNA gene. The results of these studies suggest that pregnancy has a marked effect on the vaginal microbiome, leading to greater stability, increased *Lactobacillus* proportional abundance and reduced richness and diversity relative to the vaginal microbiomes of non-pregnant women^29–33^.

The objectives of this study were to describe the vaginal microbiome of pregnant women at low risk for preterm birth and to compare their microbial profiles to those of healthy non-pregnant women^34^. This approach enabled direct comparisons between the two cohorts in which the main difference was their pregnancy status. Microbiome profiling was based on sequencing of the *cpn*60 universal target, which provides higher resolution than 16 S rRNA variable regions^35^ and has allowed us to resolve previously undescribed vaginal microbiome community state types^34^.

## Results

### Cohort description

Socio-demographic characteristics were comparable between pregnant and non-pregnant participants in terms of age, BMI, ethnicity and smoking status (Table 1). There was no significant difference in BMI (t-test, p = 0.211) or ethnicity (Chi-square, p = 0.372). However, pregnant women (age 33 ± 4) were on average older than non-pregnant women (age 31 ± 7) (t-test, p < 0.0001). The number of current smokers also differed between pregnant (2%) and non-pregnant women (12%) (t-test, p < 0.0001).

**Table 1:** Sociodemographic, clinical and microbiological characteristics across pregnant and non-pregnant cohort.

Characteristics	Pregnant	Non-pregnant
**Age** ^1,^*	(n = 182)	(n = 310)
(Mean ± SD, Range)	33.6 ± 4.3 (21–45)	30.1 ± 7.6 (18.6–49.2)
18–25	5 (2.7%)	111 (36%)
26–35	122 (67%)	131 (42%)
36–49	55 (30.2%)	68 (22%)
**Body mass index** ^1^	(n = 182)	(n = 307)
(Mean ± SD, Range)	23.1 ± 4.21 (17–43)	23.9 ± 5.2 (15–50)
Underweight (<18.50)	7 (3.8%)	18 (5.8%)
Normal weight (18.51–24.9)	140 (76.9%)	194 (62.6%)
Overweight (25.0–29.9)	26 (14.3%)	55 (17.7%)
Obese (>30)	9 (4.9%)	40 (12.9%)
**Ethnicity** ^2^	(n = 182)	(n = 306)
White	117 (64.3%)	200 (64.5%)
East Asian	26 (14.3%)	60 (19.4%)
South Asian	13 (7.1%)	12 (3.9%)
Black	4 (2.2%)	9 (2.9%)
Other/Mixed ethnicity	22 (12%)	25 (8%)
**Smoking** ^2,^*	(n = 182)	(n = 310)
Yes	4 (2.2%)	39 (12.6%)
**Alcohol**	(n = 182)	(n = 310)
Yes	11 (6%)	no data
**Unprotected sex** ^2,^*	(in the past 4 days)	(in the past 2 days)
Yes	47 (25.8%)	45 (14.5%)
**Nugent category** ^2,^*	(n = 172)	(n = 307)
Not consistent with BV	111 (61%)	250 (80.6%)
Intermediate BV	36 (20%)	25 (8%)
Consistent with BV	25 (14%)	32 (10.3%)
**CST** ^2,^*	(n = 182)	(n = 310)
I	56 (30.7%)	156 (50.3%)
II	12 (6.6%)	0 (0%)
III	30 (16.5%)	50 (16.1%)
IVA	0 (0%)	36 (11.6%)
IVC	26 (14.3%)	22 (7.1%)
IVD	42 (23.0%)	24 (7.7%)
V	16 (8.8%)	22 (7.1%)
**Estimated bacterial load (total copies of 16 S rRNA gene)/swab** ^1,^*	(n = 181)	(n = 309)
(Mean ± SD, Range)	7.77 ± 0.93 (4.89–10.67)	6.83 ± 1.55 (3.50–10.31)
10^4^ or less	1 (0.5%)	47 (15.2%)
10^5^–10^6^	27 (15%)	97 (31.4%)
10^7^–10^8^	133 (73.5%)	146 (47.2%)
10^9^ or more	20 (11%)	19 (6.1%)
**Presence of Mollicutes** ^2,^*	(n = 182)	(n = 310)
Yes	74 (40%)	217 (70%)
**Presence of** ***Ureaplasma*** ^2,^*	42 (23%)	149 (48%)
*U*. *parvum*	39 (21.4%)	127 (41%)
*U*. *urealyticum*	3 (1.6%)	5 (1.6%)
Both	0 (0%)	1 (0.3%)

^1^t-test; ^2^Chi-square. *Characteristic with significant differences (at 5% level) between pregnant and non-pregnant cohorts.

Sixty-four percent of women described themselves as of Caucasian (white) background in the pregnant cohort, which was not different from the non-pregnant cohort. This was followed by East Asian (14%) and South Asian (7%). Ten percent of participants had other or mixed ethnicity. Pregnancy outcome data was not available for five women (5/182) since they delivered in different hospitals. Sixty women (33%) were nulliparous and the average gestational ages at enrolment and at delivery were 13^+2^ and 39^+2^ weeks, respectively (Table 2). Seventeen women (9.3%) had assisted conception and the majority of women had a vaginal delivery (74%) as opposed to C-section (26%). All infants were liveborn and seven were delivered prior to 37 weeks gestation. Average birth weight was 3376 g and only three newborns weighed less than 2500 g at birth, with one of these being preterm (<37 weeks gestation). Vaginal microbiomes from the women who delivered infants less than 2500 g were determined to belong to CST II (*L*. *gasseri* dominated) (1/3) and III (*L*. *iners* dominated) (2/3). Five infants were admitted to a level 3 intensive-care unit (NICU), four of which were preterm. None of these five infants had low birth weight. Vaginal microbiomes of the mothers of the five infants were determined to belong to CST III (*L*. *iners* dominated) (1/5), V (*L*. *jensenii* dominated) (2/5), IVC (*G*. *vaginalis* subgroup A dominated) (1/5) and IVD (mixed microbiome) (1/5).

**Table 2:** Pregnant cohort description and pregnancy outcomes.

Characteristics	Descriptive
**Folic acid**	(n = 182)
Before conception	49 (26.9%)
During pregnancy	50 (27.5%)
**Vitamins**	(n = 182)
Before conception	100 (54.9%)
During pregnancy	174 (95.6%)
**Natural conception**	(n = 182)
No	18 (9.9%)
**Parity**	(n = 182)
0	60 (33%)
1	98 (53.8%)
2–4	24 (13.2%)
**Pre existing medical condition**	(n = 182)
Hypo/Hyperthyroidism	16 (8.8%)
Depression	9 (4.9%)
Asthma	6 (3.3%)
Anemia	5 (2.7%)
Other condition	41 (22.5%)
No	105 (57.7%)
**Surgeries in the past 10 years**	(n = 182)
Yes	83 (45.6%)
**Antibiotics at enrolment (for other infections excluding vaginitis/BV)**	(n = 182)
Amoxicillin/Penicillin	8 (4.4%)
Nitrofurantoin	3 (1.6%)
Other	3 (1.6%)
Unknown/Unsure	6 (3.3%)
No	162 (89%)
**Gestational age at enrollment**	(n = 182)
(Mean ± SD, Range)	13^+2^ ± 1^+1^ (11^+1^–16^+6^)
**Gestational age at delivery**	(n = 177)
(Mean ± SD, Range)	39^+2^ ± 1^+2^ (32^+1^–41^+2^)
**Preterm birth (<37 weeks)**	(n = 177) 7 (4%)
**Mode of delivery**	(n = 177)
Vaginal delivery	131 (74.0%)
C-section	46 (26%)
**Fetal sex**	(n = 177)
Female	91 (51%)
Male	86 (49%)
**Birth weight (g)**	(n = 177)
(Mean ± SD, Range)	3376 ± 474 (1876–5200)
Low (<2500 g)	3 (1.7%)
Normal (2500–4200 g)	166 (93.8%)
Large (>4200 g)	8 (4.5%)
**Apgar score at 5 min**	(n = 177)
(Mean ± SD, Range)	8.97 ± 0.17 (8–9)
**Neonate in level 3 care nursery after birth**	(n = 176) 5 (2.8%)

### Sequencing results and OTU analysis

We characterized the vaginal microbiomes of pregnant women at low risk of preterm birth using pyrosequencing of the universal target region of the *cpn*60 gene. Sequence reads from the vaginal microbiomes of pregnant women were mapped on to a manually curated reference set of 1,561 OTU sequences as described in the methods. Raw sequence data files for the 182 samples described in this study were deposited to the NCBI Sequence Read Archive (BioProject PRJNA317763). A total of 1,415,117 *cpn*60 reads was generated. Median and average read count per sample was 5,024 and 7,775 (range 494–43,245), respectively. Average read length was 448 bp. The average MAPQ value was 21.1.

Results of Bowtie2 mapping showed that these reads corresponded to 645 OTUs from the reference assembly (Supplementary Table S1). A total of 82 OTUs (corresponding to 53 nearest neighbour “species”) were at least 1% abundant in at least one sample. And only 22 “species” were detected in at least 50% of samples (Table 3). Although the ranges of percent identity to reference sequences are large in some cases, reflecting the diversity in the community, the most abundant OTU were at the high end of the range. In fact, of the 25 most abundant OTU in the study (accounting for 95% of the sequence reads generated), 22/25 were >95% identical to their nearest neighbour (Supplementary Table S1).

**Table 3:** Prevalence and proportion of total reads for “species” detected in at least 50% of samples.

Nearest neighbour^a^	% OTU identity range	Prevalence/182 (%)	% total reads
*Lactobacillus jensenii*	81.6–100	181 (99.4)	10.66
*Streptococcus devriesei*	83	180 (98.9)	0.34
*Lactobacillus crispatus*	78.8–99.8	178 (97.8)	31.93
*Lactobacillus acidophilus*	91.8–100	178 (97.8)	1.22
*Atopobium vaginae*	82.5–96.9	176 (96.7)	1.62
*Weissella viridescens*	58.8–99.5	173 (95.0)	0.22
*Desulfotalea psychrophila*	76.1	167 (91.7)	0.12
*Lactobacillus iners*	86.8–100	165 (90.6)	15.85
*Lactobacillus gasseri*	65.4–100	164 (90.1)	5.69
*Streptococcus parasanguinis*	94.9–97.0	162 (89.0)	0.08
*Prevotella tannerae*	77.5–98.7	162 (89.0)	0.07
*Faecalibacterium cf*. *prausnitzii*	77.5–79.6	159 (87.3)	0.16
*Gardnerella vaginalis* subgroup C	78.5–99.3	156 (85.7)	4.45
*Peptoniphilus harei*	89.9–98.5	156 (85.7)	0.07
*Clostridium innocuum*	75.4	148 (81.3)	0.06
*Sphingobium yanoikuyae*	99.3–99.4	145 (79.6)	0.08
*Eubacterium siraeum*	84.4	144 (79.1)	0.05
*Gardnerella vaginalis* subgroup B	93.2–99.6	133 (73.0)	0.54
*Massilia timonae*	82	126 (69.2)	0.03
*Gardnerella vaginalis* subgroup A	86.7–99.6	123 (67.5)	7.64
*Bifidobacterium breve*	89.6–99.5	119 (65.3)	2.12
*Megasphaera* sp. genomsp. type 1	58.1–86.7	112 (61.5)	3.09

^a^Closest match in the cpnDB reference database based on sequence identity.

Most reads (68.8%) were identified as *Lactobacillus* spp. And only three OTUs, all of which matched to *Lactobacillus* spp., accounted for 55.7% of all reads generated: OTU 1403: *L*. *crispatus* (30.6%), OTU 1174: *L*. *iners* (15.4%) and OTU 1479: *L*. *jensenii* (9.7%). Species with the highest sample prevalence were *L*. *jensenii* (181/182), *Streptococcus devriesei* (180/182), *L*. *crispatus* (178/182), *L*. *acidophilus* (178/182), followed by *Atopobium vaginae* (176/182) and *Weissella viridescens* (173/182). Even though OTUs with similarity to *Streptococcus devriesei* and *Weissella viridescens* were detected in most samples, they had very low abundance, representing only 0.33% and 0.22% of all reads, respectively (Supplementary Table S1).

### Community state type analysis

**Figure 1:**
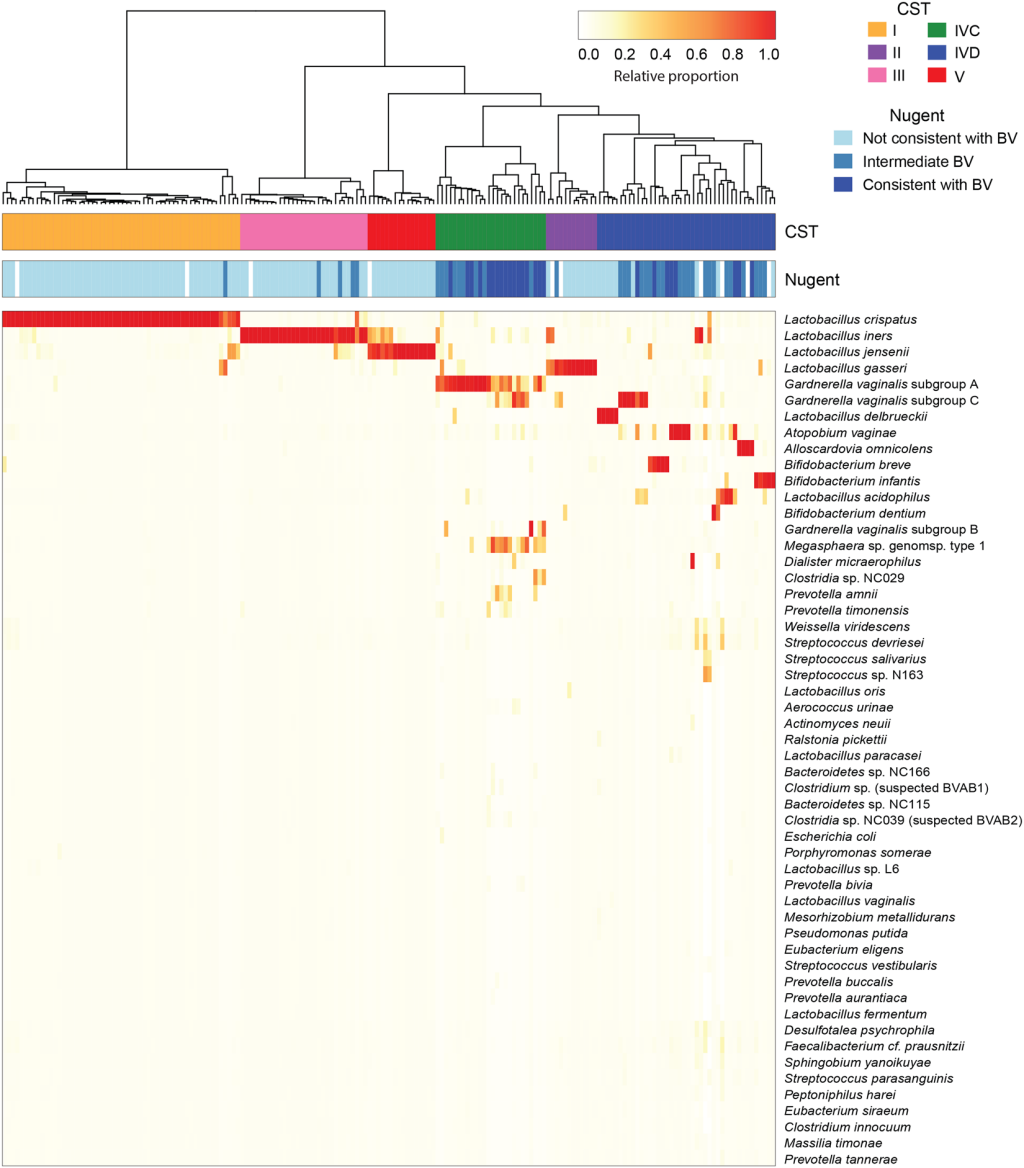
Vaginal microbial profiles of pregnant women at low risk of preterm birth. Heatmap of hierarchical clustering of Jensen-Shannon distance matrices with Ward linkage on the relative proportions of reads for each OTU within individual vaginal samples (n = 182). Each column represents a woman’s vaginal microbiome profile, and each row represents an OTU. Only OTU that are at least 1% abundant in at least one sample are shown. The proportion of the total microbiome comprised is indicated by white to red colour according to the legend. The coloured bars above the heatmap show the community state type (CST) and the Nugent score category (Nugent) for each woman. Legend: white = missing data.

Hierarchical clustering of vaginal microbiome profiles from pregnant women resulted in the resolution of six Community State Types (CST) (Fig. 1). All *Lactobacillus*-dominated CST, previously described by Ravel and Gajer^36^ based on pyrosequencing of the 16 S rRNA gene, were detected. Most profiles (114/182) were dominated by one of four *Lactobacillus* species that define four different CST: CST I (*L*. *crispatus*, n = 56), CST II (*L*. *gasseri*, n = 12), CST III (*L*. *iners*, n = 30), and CST V (*L*. *jensenii*, n = 16).

All non-*Lactobacillus*-dominated samples were assigned to CST IVC or CST IVD, as previously described by Albert *et al*.^34^. CST IVC (n = 26) is dominated by *Gardnerella vaginalis* subgroup A, *Megasphaera* sp. *genomosp* type 1, and *G*. *vaginalis* subgroup C. CST IVD (n = 42) was the most heterogeneous group, including a mixture of *Bifidobacterium dentium*, *B*. *infantis*, *B*. *breve*, *L*. *delbrueckii*, *Alloscardovia omnicolens*, *G*. *vaginalis* subgroup C, and *Atopobium vaginae*.

CST distribution of the pregnant participants was compared to the previously described non-pregnant cohort. Twelve pregnant women had microbiome profiles identified as the *L*. *gasseri*-dominated CST II, which was not observed among the profiles of non-pregnant women^34^. Additionally, CST IVA was not detected among pregnant women, including 61 with intermediate or high Nugent scores. In the non-pregnant group, 14/57 (24.6%) of women with intermediate or high Nugent scores were assigned to CST IVA, with the remainder in CST IVC (15/57, 26.3%), CST IVD (20/57, 35.1%) or one of the *Lactobacillus* dominated CST (8/57, 14.0%). CST IVA was defined by Albert *et al*.^34^ as a very heterogeneous group, dominated by *G*. *vaginalis* subgroup B or *Atopobium vaginae*, or combinations of *Stapylococcus*, *Streptococcus*, *Prevotella*, *Alloscardovia*, *Gardnerella*, *Bifidobacterium* and *Lactobacillus*.

**Figure 2:**
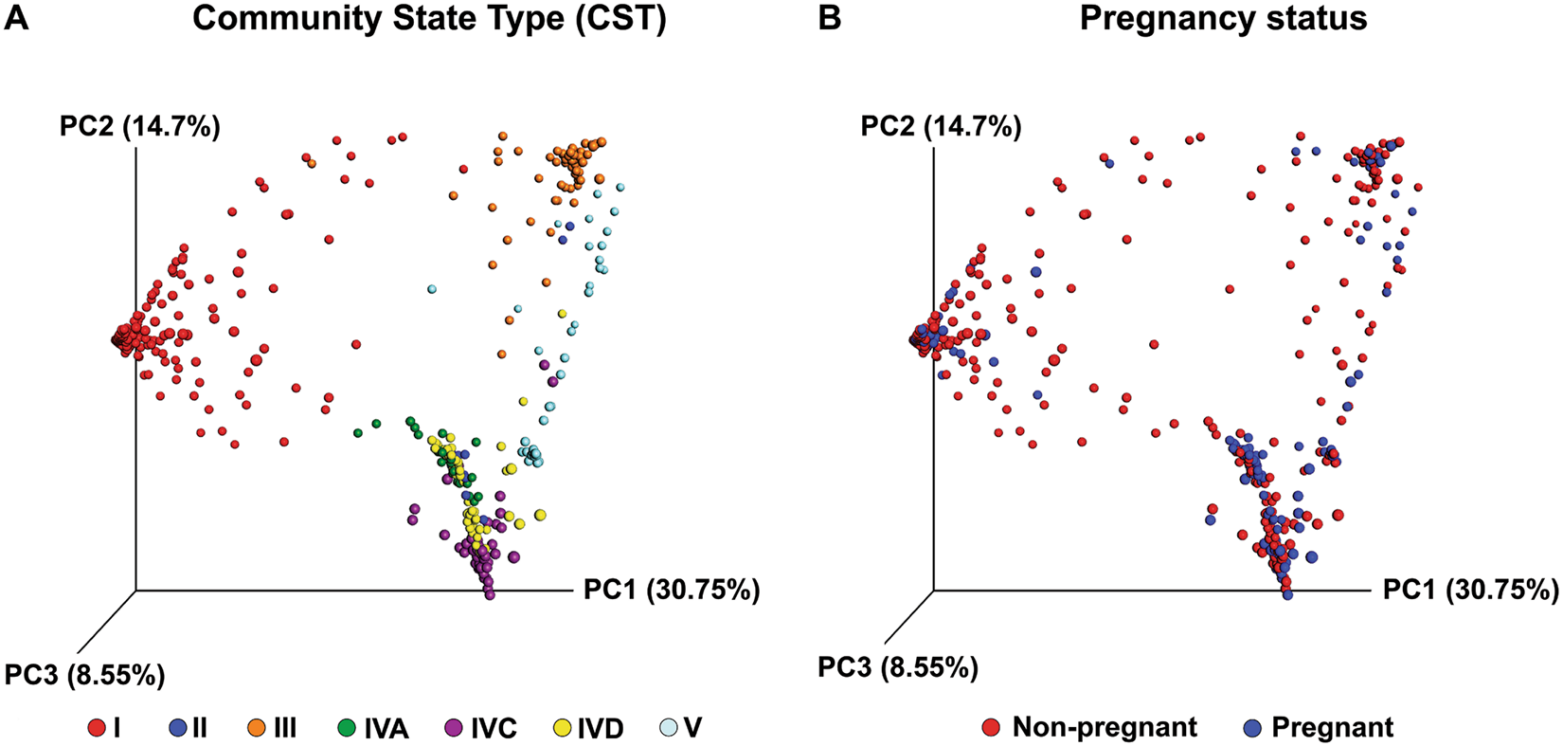
CST and pregnancy status of participants. Jackknifed principal coordinates analysis (PCoA) of Bray-Curtis distance matrices of microbial profiles from all participants in the study, with individuals coloured by CST (**A**) or pregnancy status (**B**). Samples with fewer than 1000 sequence reads (16/492) were not plotted.

As expected, *Lactobacillus*-dominated CST, i.e. CST I, CST II, CST III and CST V, were associated with low Nugent score samples (BV negative), while CST IVC and CST IVD were associated with intermediate and high Nugent scores (BV positive) (Fig. 1). Although there were differences regarding presence/absence of specific CST in the pregnant and non-pregnant cohorts, overall microbial profiles could not be distinguished based on pregnancy status alone (Fig. 2A,B).

### Abundance of *Lactobacillus* spp

**Figure 3:**
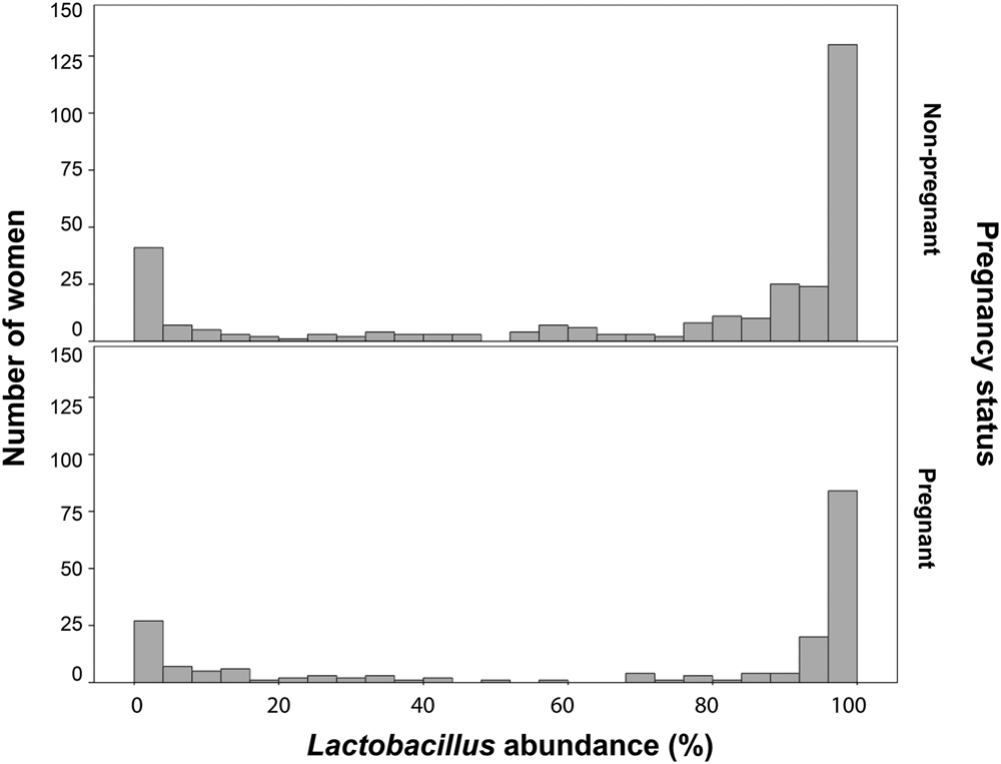
*Lactobacillus* spp. abundance. Bimodal distribution of vaginal microbiome profiles of non-pregnant (upper panel) and pregnant (lower panel) women based on *Lactobacillus* spp. abundance (%).

**Figure 4:**
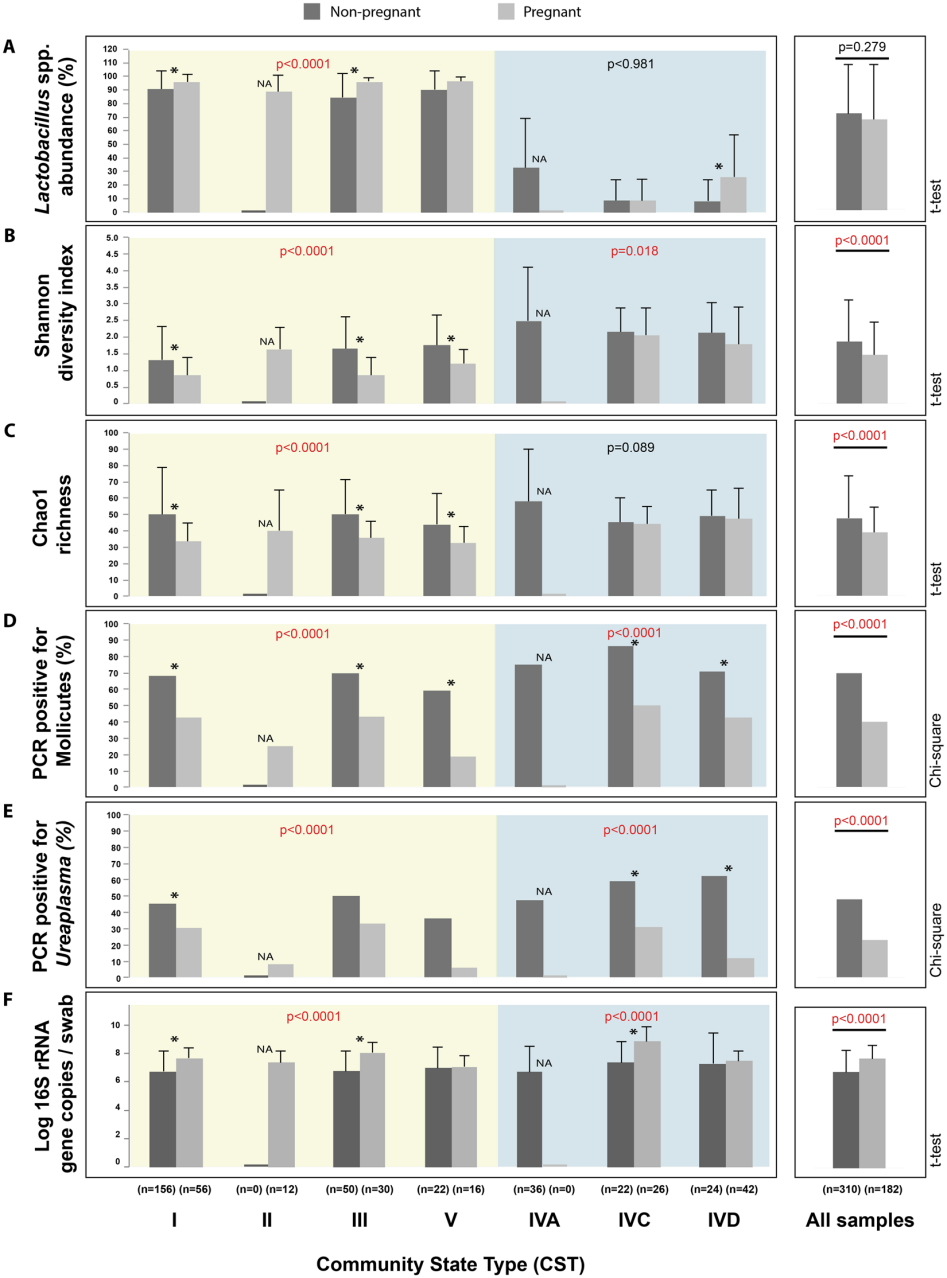
Comparison of the microbial community features between pregnant and non-pregnant participants within each CST. *Lactobacillus* spp. abundance (**A**), Shannon diversity (**B**), Chao1 richness (**C**), Mollicutes prevalence (**D**), *Ureaplasma* prevalence (**E**) and bacterial load (**F**) were compared between pregnant and non-pregnant women in each CST. For continuous variables (**A**–**C**,**F**), the mean value is plotted with error bars indicating standard deviation. Significant differences (p < 0.05) between pregnant and non-pregnant women within each CST are indicated by an asterisk. p-values in the main panels refer to the comparison between pregnant and non-pregnant women in *Lactobacillus*-dominated CST (yellow panel) and non-*Lactobacillus*-dominated CST (blue panel). A comparison of pooled data from all CST is shown in the right-most panel. Statistical tests used are indicated on the right side of the graph.

The proportion of *Lactobacillus* spp. in vaginal samples followed a bimodal distribution (Fig. 3). Microbiomes of most pregnant women had either low (0–20%, n = 46/182) or high (80–100%, n = 113/182) *Lactobacillus* spp. abundance, whereas only 23/182 (12.6%) of samples had intermediate levels (20–80%) of *Lactobacillus* spp. Also, pregnant women in *Lactobacillus*-dominated CST had greater proportions of lactobacilli (95.7% ± 6.2) (i.e. were more *Lactobacillus* dominated) than non-pregnant women (89.9% ± 14.8) (t-test, p < 0.0001) (Fig. 4A).

### Ecological analysis

Assessment of alpha diversity revealed that microbiomes of pregnant women were less diverse (Shannon diversity index, 1.3 ± 0.9) and less rich (Chao1, 38.9 ± 15.3) when compared to those of non-pregnant women (1.6 ± 1.1; 47.6 ± 26) (t-test, p < 0.001) (Fig. 4B,C). When comparisons were conducted within CST, profiles of pregnant women in CST I, III and V were less diverse and less rich than profiles of non-pregnant women in the same category (t-test, p < 0.05) but no statistically significant differences were observed in CST IVC and IVD.

### Prevalence of Mollicutes and *Ureaplasma* (PCR)

Mollicutes (*Mycoplasma* and/or *Ureaplasma*) were detected by family-specific conventional PCR in 74/182 (40%) of pregnant women, but prevalence varied between CST: 43%, 25%, 43%, 18%, 50% and 43% in CST I, II, III, V, IVC and IVD, respectively (Fig. 4D). *Ureaplasma* species were detected by genus-specific PCR in samples of 42/182 (23%) pregnant women, with 39 testing positive for *U*. *parvum* and 3 for *U*. *urealyticum* (Fig. 4E). All pregnant women PCR positive for *U*. *urealyticum* (3/3) were in CST III.

Pregnant women were less likely to be positive for Mollicutes detection by PCR when compared to non-pregnant women, regardless of CST (Chi-square, p < 0.0001, Mollicutes positive: 217/310 non-pregnant women and 74/182 pregnant women) (Fig. 4D). Additionally, pregnant women in both *Lactobacillus*- and non-*Lactobacillus*-dominated CST had lower prevalence of *Ureaplasma* species than non-pregnant women (Chi-square, p < 0.0001, *Ureaplasma* positive: 149/310 non-pregnant women and 42/182 pregnant women) (Fig. 4E).

### Total bacterial load (qPCR)

Total bacterial load was assessed based on qPCR targeting the 16 S rRNA gene, and expressed as log copy number per swab (Fig. 4F). Average bacterial load was log 7.77 ± 0.93 16 S rRNA gene copies per swab (range log 4.89–10.67). Bacterial load within each CST for the pregnant women’s samples were: CST I (log 7.6 ± 0.7), CST II (log 7.3 ± 0.8), CST III (log 8.0 ± 0.7), CST V (log 7.0 ± 0.7), CST IVC (log 8.8 ± 1) and CST IVD (log 7.4 ± 0.7).

Higher bacterial loads were detected in samples from pregnant women (log 7.7 ± 0.9) when compared to non-pregnant women (log 6.8 ± 1.5) (t-test, p < 0.0001) (Fig. 4F). Comparisons within CST confirmed that samples from pregnant women in CST I (log 7.6 ± 0.7), CST III (log 8.0 ± 0.7) and CST IVC (log 8.8 ± 1) had higher bacterial load than non-pregnant women in the same categories (CST I = log 6.7 ± 1.4, CST III = log 6.7 ± 1.4. CST IVC = log 7.3 ± 1.5) (t-test, p < 0.0001). In a second analysis, samples were pooled into two groups: *Lactobacillus*- (I, II, III, V) and non-*Lactobacillus* dominated (IVA, IVC, IVD) CST. Bacterial load values were statistically different (t-test, p < 0.0001) between pregnant and non-pregnant women in both groups, with pregnant women having greater bacterial load (*Lactobacillus*-dominated CST: log 7.6 ± 0.8, non-*Lactobacillus* dominated CST: log 8 ± 1) than non-pregnant women (*Lactobacillus*-dominated CST: log 6.7 ± 1.4, non-*Lactobacillus* dominated CST: log 7.0 ± 1.8).

### Relationships between microbiological and socio-demographic characteristics across the pregnant cohort

**Figure 5:**
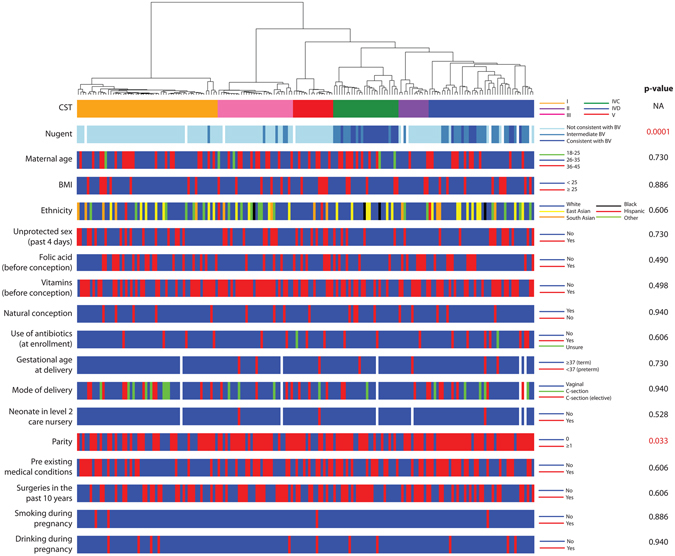
Socio-demographic characteristics of pregnant participants in relation to CST. Hierarchical clustering of microbiome profiles based on Jensen-Shannon distance matrices with Ward linkage of the relative proportions each OTU within individual vaginal samples (n = 182). Demographic characteristics are indicated on the left side, and categories indicated on the right side of each row. Numbers on the right side indicate adjusted p-values of Chi-square test after false discovery rate correction. Fisher’s Exact test was conducted for variables where at least one category had an expected frequency of less than 5. Legend: white = missing data.

The characteristics of the microbial community of pregnant women were analyzed in terms of their relationship with the socio-demographic and clinical data. First, we determined whether there was any relationship between the CST (I, II, III, IVC, IVD, V) and demographic characteristics such as BMI, ethnicity, unprotected sex, folic acid intake, vitamins, natural conception, antibiotic use, gestational age at delivery, mode of delivery, neonatal in high level care nursery, parity, pre-existing conditions, surgeries (past 10 years), smoking and alcohol drinking status as well as Nugent score (Fig. 5). Besides Nugent score, the only significant interaction was between CST and parity (0 or ≥ 1) (Chi-square, p = 0.033), with 45% of women at parity 0 in CST I (27/60) and 23% of women at parity ≥ 1 in CST I (29/122). Microbiological and demographic characteristics were also compared to presence of Mollicutes (yes/no) and *Ureaplasma* (yes/no), microbiome richness (continuous variable) and diversity (continuous variable). These four observations were compared to 29 other variables (Supplementary Methods). PCR detection of Mollicutes (p = 0.017) and *Ureaplasma* (p = 0.017) was significantly associated with bacterial load (Chi-square).

Although smoking has been previously shown to have a possible effect on the vaginal microbiota^37^, there was no significant association between CST and smoking status in this study (Chi-square, p > 0.05). Also, microbiome analysis was redone excluding samples from participants who are smokers (results not shown). The results led to the same conclusions regarding the overall microbiome (PCoA), *Lactobacillus* abundance, Shannon diversity, Chao1 richness, bacterial load and Mollicutes/*Ureaplasma* prevalence.

## Discussion

In this study we characterized the vaginal microbiome of pregnant women with healthy ongoing pregnancies, at low risk of experiencing pregnancy complications such as preterm birth, and compared these results to our previously characterized cohort of non-pregnant women of similar ethnicity. We focussed our studies on the vaginal microbiome at 11–16 weeks. Pregnancy pathologies such as spontaneous preterm birth and early onset pre-eclampsia have their origins in the first or early second trimester and therapeutic interventions at this stage have been shown to be efficacious^38^. This is also the gestational age at which pregnant women in Canada often have their first prenatal visit with a health care provider and a vaginal swab is taken to assess the presence or absence of vaginal/cervical infections.

We used the *cpn*60 universal target region in our study due to its superior resolution of bacterial taxa relative to 16 S rRNA sequences^39–45^, its establishment as a preferred barcode for bacteria^35^, and the availability of a reference database of vaginal bacterial *cpn*60 sequences from previous studies^34, 46–49^. This approach has been applied to numerous animal^50–52^, human^53^ and environmental microbial populations^54–57^ and has previously resulted in discovery of novel community state types based on the resolution of distinct subgroups of *Gardnerella vaginalis* ^34^.

In order to make valid comparisons between pregnant and non-pregnant women microbiomes, we analysed the results from both cohorts based on socio-demographic characteristics (Table 1). The two cohorts were comparable, with no significant differences detected in any category except for maternal age and smoking. Differences regarding smoking status were not surprising since behavioural changes such as reduced drinking and smoking have been documented in pregnancy^28^. Although statistically significant, the identified difference in maternal age (pregnant: 33 ± 4 and non-pregnant: 30 ± 7) was not considered biologically relevant since the difference of the mean values was only 3 years.

Overall microbial profiles could not be distinguished from each other based on pregnancy status alone. However, a more detailed analysis revealed several differences between the microbiomes of healthy pregnant women and those of healthy non-pregnant women. The BV-associated CST IVA (dominated by *G*. *vaginalis* subgroup B and *Atopobium*) was not detected in the pregnant cohort, whereas *L*. *gasseri* dominated CST II, which was not detected among the 310 non-pregnant women in our previous study^34^, was detected in 12 women in the pregnant cohort. Pregnant women in *Lactobacillus*-dominated CST had higher relative abundance of *Lactobacillus* spp. when compared to non-pregnant women. Vaginal microbiomes of pregnant women had lower richness and diversity and a correspondingly lower prevalence of Mollicutes and *Ureaplasma* when compared to non-pregnant women.

Microbial profiles from the pregnant women clustered in six different groups, mostly *Lactobacillus-*dominated CST (CST I, CST II, CST III and CST V), originally defined by Ravel & Gajer^58^. Non-*Lactobacillus*-dominated (CST IV) profiles are described in the literature as either very heterogeneous or dominated with BV-associated bacteria^34, 59^. None of the pregnant women, including 61 with intermediate or high Nugent scores, were identified as belonging to CST IVA, which is characterized by dominance of *G*. *vaginalis* subgroup B and *Atopobium* ^34^. This distribution is notably different from the non-pregnant cohort, where 24.6% of women with intermediate or high Nugent scores were assigned to CST IVA^34^. Results of other studies have suggested that CST dominated by BV-associated microorganisms are less frequently detected in pregnancy^30, 32^. While our study design does not allow us to address the issue of overall prevalence of BV-associated CST in pregnancy, our results suggest that the distribution of these CST among pregnant women may be different than in non-pregnant women. This suggests a role CST IVA could be playing in early pregnancy loss.

OTUs that were weakly similar to *Streptococcus* and *Weissella* species were detected in most samples, but they represented only 0.33% and 0.22% of all reads, respectively. These OTUs were previously described as highly prevalent in the vaginal microbiome of healthy non-pregnant women^34^. They have low sequence identity to any reference sequences in the cpnDB_nr database (OTU 0026: *S*. *devriesei* 83% and OTU 0021: W. *viridescens* 58.8%). However, this subset of the cpnDB database contains only selected representative sequences of named species. A broader search shows that these OTU are more similar to metagenomic sequences derived from the fecal microbiome (OTU 0021 is 97% identical to Genbank accession GQ178631) or oral microbiome (OTU 0026 is 85% identical to KJ406686) that represent uncharacterized Firmicutes; reminders of the common but still uncharacterized constituents of the human microbiome.

Our findings of greater *Lactobacillus* abundance and lower richness and diversity in the vaginal microbiomes of pregnant women relative to non-pregnant women are consistent with previous studies in the literature. Aagaard *et al*.^29^ analyzed the microbiomes of 24 healthy, pregnant American women sampled at three different locations within the vagina. Vaginal site did not drive the structure of the microbial community, but the authors found that overall microbiomes of pregnant women were less diverse and less rich when compared to non-pregnant women. Similarly, Walther-António *et al*.^31^ described the microbiomes of 12 White pregnant American women based on longitudinal sampling and observed reduced microbiome diversity and higher *Lactobacillus* spp. relative abundance during pregnancy. Romero *et al*.^30^ also reported that *Lactobacillus* spp. abundance was significantly higher in pregnant women in comparison to non-pregnant and increased as a function of gestational age. They also described higher stability of the microbiome during pregnancy when compared to reproductive age non-pregnant women. In another longitudinal study, MacIntyre *et al*.^32^ analyzed the microbiomes of 42 British women during pregnancy and the post-partum period. *L*. *jensenii*-dominated profiles were more common among these women than Northern American women. The authors also observed that post-partum microbiomes become less *Lactobacillus* spp. dominant and more rich and diverse (i.e. more similar to the microbiomes of non-pregnant women) regardless of ethnicity, providing strong support for the idea that pregnancy has a transient effect on the vaginal microbial community. Importantly, the conclusions of these studies and our current study are consistent despite differences in the cohort studied (Canadian, American or European cohorts of varying mixtures of ethnicity), universal target amplified (*cpn*60 universal target or 16 S rRNA gene) or sequencing platforms used (454/Roche pyrosequencing or Illumina MiSeq).

The explanations for these pregnancy-associated changes are not well established, but a relationship between sex steroid hormone levels and the composition of the vaginal microbiome has been previously reported^58, 60, 61^. Increased levels of estrogen during pregnancy lead to increased thickness of the vaginal mucosa and increased deposition of glycogen^62, 63^. Glycogen is the main carbohydrate utilized by *Lactobacillus* spp. for the production of lactic acid, which contributes to the protective effect of a low vaginal pH^64–67^. This may contribute to the greater dominance of *Lactobacillus* in pregnancy and, consequently, the lower richness and diversity in this cohort.

A novel finding in our study was the lower prevalence of Mollicutes and *Ureaplasma* in pregnancy detected by family and genus-specific PCR. Mollicutes have been associated with preterm birth, preterm premature rupture of membranes and low birth weight^68–70^. The lower prevalence of Mollicutes and *Ureaplasma* is consistent with the overall lower species richness and diversity in the vaginal microbiomes of pregnant relative to non-pregnant women. We also found that pregnant women had higher bacterial load than non-pregnant women as estimated by quantitative PCR targeting the 16 S rRNA gene. Hormone induced production of glycogen may offer a nutritionally richer environment for bacterial growth in the vagina during pregnancy. Additionally, it is known that pregnancy alters the amount and consistency of the mucus, which becomes more abundant and thicker^71^. Thus, it is possible that swabs sampled from the pregnant women carried more material when compared to non-pregnant women. We are unable to resolve this question since the swabs were not weighed before DNA extraction steps. In addition to pregnancy associated physiological differences and mucus consistency, other technical factors such as storage conditions or inherent differences in the study populations cannot be ruled out.

One limitation of this study was the assessment of pregnancy outcomes and microbial profiles since there were very few poor outcomes in this cohort, as it would be expected for a low risk group. This study, however, does provide crucial baseline information for future studies in pregnant women. In addition, we detected significant interaction between parity and CST. Considering the large number of variables in the metadata, analysis of these associations should be interpreted with caution. We can speculate that the prevalence of the *L*. *crispatus*-dominated CST I among nulliparous women might be associated with more cautious or health conscious behaviour among women in their first gestation. Pregnancy-induced physiologic alterations that can persist after delivery have been previously reported^72^. In addition to physiologic changes, a disturbed vaginal microbiome that persisted for up to a year post-partum has also recently been described^33^. Those persistent changes might explain the association between CST and parity we observed, with post-partum microbiome changes affecting the current status of primiparous and multiparous women. Further studies are needed to investigate these relationships in more detail.

In conclusion, we have identified several differences in the composition of the vaginal microbial communities of pregnant women living in Canada relative to non-pregnant women: larger total bacterial community, lower richness and diversity, higher *Lactobacillus* abundance and lower Mollicutes/*Ureaplasma* prevalence. These findings give us a better understanding of the vaginal microbiome in pregnancy, which is a critical step toward being able to exploit the diagnostic potential of the microbiome for the prediction of adverse pregnancy outcomes as well as to explore alternative therapeutic procedures through microbiological intervention. Establishing an understanding of the normal microbiome in low risk pregnant women is a vital baseline for comparison to the microbiome of women who have adverse perinatal outcomes such as preterm birth.

## Methods

### Study population and sampling

This study received ethical approval from the Mount Sinai Hospital Research Ethics Board (Approval Number 08-0005-A). All participants provided written informed consent and all methods were performed in accordance with the relevant guidelines and regulations. Women attending antenatal clinics at Mount Sinai Hospital (Toronto, ON, Canada) between May 2012 and October 2013 were invited to be part of a clinical trial to determine the effect of oral probiotic lactobacilli in altering the vaginal microbiome in asymptomatic pregnant women with an abnormal Nugent score^73, 74^. Nugent score was determined on Gram-stained swabs taken at the same time as the swab for microbial analysis. Women with normal Nugent scores were excluded from the probiotic trial and are included in this analysis. Samples from women with an abnormal Nugent score who were subsequently randomized and included in the analysis for this study were taken prior to any intervention in these women and there was no difference in the pregnancy outcomes between the lactobacilli and placebo groups^74^. A preliminary report on the results of that probiotic trial have been presented previously^74^.

Women were eligible to participate if the following inclusion criteria were met: currently pregnant, adequate comprehension of English language to sign written informed consent, age ≥ 18 years old, no evidence of fetal complications such as intrauterine growth restriction, and no evidence of medical complications of pregnancy. Exclusion criteria included inability to provide informed written consent, multi-fetal pregnancies, currently taking antibiotics or other antimicrobial therapy for BV treatment. Study data were collected and managed using REDCap electronic data capture tools^75^.

Vaginal swabs were collected under direct visualization using a speculum by either a physician or a nurse and placed in dry tubes prior to being placed in −80 °C. A total of 182 pregnant women at 11–16 weeks gestation were enrolled in the vaginal microbiome study, including 111 women with normal Nugent scores (inconsistent with BV), 61 women with Nugent scores that were intermediate or consistent with BV, and 10 women with indeterminate Nugent scores due to poor quality smears. Total nucleic acid was extracted from swabs using the MagMAX™ Total Nucleic Acid Isolation Kit (Life Technologies, Burlington, ON, Canada) as per manufacturer’s instructions. Kit reagents are aliquoted to eliminate repeated accessing of open reagents, and samples are processed in small batches using filter-tips to prevent cross-contamination. Pipettes and other lab surfaces are regularly treated with DNA surface decontaminant (DNA Away, ThermoFisher Scientific, Waltham, MA). Regular monitoring of reagent only DNA extraction controls in our lab by universal PCR confirms that these procedures are sufficient to eliminate detectable template contamination of study samples.

The microbial profiles of low-risk pregnant women were compared to profiles previously generated from healthy, reproductive aged, non-pregnant Canadian women from the greater Vancouver area, British Columbia, Canada (n = 310)^34^. Samples were collected as being non-menstrual but were not sampled at any specific non-menstrual cycle time as other studies have demonstrated there is little variation in microbiome profiles through the cycle^48^. Samples from this previous study were processed in the same way as in the current work in terms of swab type, storage temperature, DNA extraction, library preparation and sequencing. Although the year of sampling was different between the two cohorts, there was no difference in time from sample collection to sequencing between the two groups.

### Total Bacterial DNA (qPCR) and Detection of Mollicutes (PCR)

*Quantitative PCR* (*qPCR*)*:* Total bacterial DNA quantity in each sample was estimated using a SYBR Green assay based on amplification of the V3 region of the 16 S rRNA gene. Primer sequences were as follows: SRV3-1 (5′-CGGYCCAGACTCCTAC-3′), SRV3-2 (5′-TTACCGCGGCTGCTGGCAC-3′)^76^. Reactions run on a MyiQ thermocycler using the following cycling parameters: 95 °C for 3 min, followed by 30 cycles of 95 °C for 15 sec., 62 °C for 15 sec., 72 °C for 15 sec., with a final extension at 72 °C for 5 minutes^77^.

*Conventional PCR:* Some Mollicutes (*Mycoplasma* and *Ureaplasma*) species lack a *cpn*60 gene^78^. Thus, we performed a family-specific semi-nested PCR targeting the 16 S rRNA gene to detect Mollicutes^79^, and a PCR targeting the multiple banded antigen gene to detect *Ureaplasma* spp.. In this assay, PCR products from *Ureaplasma parvum* and *U*. *urealyticum* can be differentiated by size^80^.

### *cpn*60 Universal Target (UT) PCR and Pyrosequencing

Universal primer PCR targeting the 552–558 bp *cpn*60 UT region was performed using a mixture of *cpn*60 primers consisting of a 1:3 molar ratio of primers H279/H280:H1612/H1613, as described previously^47, 48, 81^. To avoid cross-contamination, samples were handled in small batches, and a no template control was included with each set of PCR reactions. To allow multiplexing of samples in a single sequencing run, primers were modified at the 5′ end with one of 24 unique decamer multiplexing identification (MID) sequences, as per the manufacturer’s recommendations (Roche, Brandford, CT, USA). Amplicons were pooled in equimolar amounts for sequencing on the Roche GS Junior sequencing platform. The sequencing libraries were prepared using the GS DNA library preparation kit and emulsion PCR (emPCR) was performed with a GS emPCR kit (Roche Diagnostics, Laval, Canada).

### Analysis of Operational Taxonomic Units (OTU)

Raw sequence data was processed by using the default on-rig procedures from 454/Roche. Filter-passing reads were used in the subsequent analyses for each of the pyrosequencing libraries. MID-partitioned sequences were mapped using Bowtie 2 (http://bowtie-bio.sourceforge.net/bowtie2/) on to a manually curated reference set of 1,561 OTU sequences representing human vaginal microbiota. Bowtie 2 was run using the default end-to-end alignment mode, in which the minimum “cutoff” for any individual read to be validly aligned to a reference sequence is an alignment score of −0.6 + −0.6 * L, where L is the length of the read. The best valid alignment for each read is reported. Mapping quality was also evaluated by MAPQ value, which is based on the probability that alignment does not correspond to the read’s true point of origin.

The OTU reference set was generated originally by *de novo* assembly of *cpn*60 sequence reads from each of 546 vaginal microbiomes, which included 182 samples from pregnant women (this study) and 364 samples from non-pregnant women from previous studies by our research group. The reference assembly was created by the microbial Profiling Using Metagenomic Assembly pipeline (mPUMA, http://mpuma.sourceforge.net)^82^ with Trinity as the assembly tool^83^. Assembled OTU were labeled according to their nearest reference sequence determined by watered-Blast comparison^84^ to the cpn60 reference database, cpnDB_nr (downloaded from http://www.cpndb.ca ^78^). cpnDB_nr is a subset of the cpnDB database that includes a non-redundant collection of sequences representing all species in cpnDB, with a preference for inclusion of the type strain for each species when available. This reference assembly approach allows us to compare the microbial profiles from various cohorts under investigation, including the 182 pregnant women described in this study. To improve comprehension of some figures, we have pooled reads from OTU into “nearest neighbour species” based on their taxonomic label. Thus, the term “species” refers to OTUs that have the same nearest neighbour match in cpnDB.

### Statistical Analysis

Comparisons across pregnancy status cohorts were based on analysis of variance (ANOVA), t-test and Chi-square, performed in IBM SPSS (Statistical Package for the Social Sciences, version 21) at 5% level of significance. For analysis of associations between socio-demographic characteristics and microbiome profiles, a false discovery rate (FDR) correction for multiple comparisons was applied^85^ (for the complete list of variables tested, see Supplementary Methods).

Alpha (Shannon diversity and Chao1 estimated species richness) and beta diversity (jackknifed Bray-Curtis dissimilarity matrices) were calculated as the mean of 100 subsamplings of 1000 reads (or all reads available when less than 1000) in QIIME (Quantitative Insights Into Microbial Ecology)^86^. Plots of alpha diversity measures against bootstrap sample number were generated in R and visually inspected to ensure that an adequate sampling depth for each sample was achieved. Microbiome profiles were also compared based on Bray-Curtis dissimilarity matrices using Principal coordinates analysis (PCoA) in QIIME.

For community state type analysis, a Jensen-Shannon distance matrix was calculated using the ‘vegdist’ function in the *vegan* package^87^ with a custom distance function that calculates the square root of the Jensen-Shannon divergence^88^. This distance matrix was used for hierarchical clustering using the ‘hclust’ function in R, with Ward linkage.

### Data Availability

Raw sequence data files for the 182 samples described in this study were deposited to the NCBI Sequence Read Archive (Accession SRP073152, BioProject PRJNA317763). Due to ethical and legal restrictions related to protecting participant privacy imposed by the Mt. Sinai Hospital Research Ethics Board, all other relevant data are available upon request pending ethical approval. Please submit all requests to initiate the data access process to the corresponding author.

## Electronic supplementary material

Supplementary Information

Supplementary Table S1
