# Supplementary material for: The vaginal microbiome of pregnant women is less rich and diverse, with lower prevalence of Mollicutes, compared to non-pregnant women
Source: Sci Rep. 2017 Aug 23;7:9212. doi: 10.1038/s41598-017-07790-9 (PMC5569030; doi:10.1038/s41598-017-07790-9)
Supplement: Supplementary file 1 — Supplementary Information [file 41598_2017_7790_MOESM1_ESM.pdf]

Supplementary information

The vaginal microbiome of pregnant women is less rich and diverse, with lower prevalence of Mollicutes, compared to non-pregnant women

Aline C. Freitas[1], Bonnie Chaban[1#], Alan Bocking[2,3^], Maria Rocco[3], Siwen Yang[2,3], Janet E. Hill[1^], Deborah M. Money[4,5^*], and the VOGUE Research Group

Supplementary Methods

Comparison between microbiological and demographic characteristics of the pregnant cohort.

Microbiological and demographic characteristics were compared to CST (I, II, III, IVC, IVD, V), presence of Mollicutes (yes/no) and *Ureaplasma* (yes/no), microbiome richness (continuous variable) and diversity (continuous variable). Each of these five observations were compared to the following metadata: Nugent score category (BV-, intermediate BV, BV+), 16S RNA gene copy number ($<10^4$, 10^5-10^6, 10^7-10^8, $>10^9$), maternal age (18-25, 26-35, 36-45), maternal age (18-35, 35-45), BMI category (underweight, normal, overweight, obese), BMI (<25, ≥25), ethnicity (White, East Asian, South Asian, Black, Hispanic, Other), ethnicity (White, East Asian, South Asian, Other), ethnicity (White, Asian, Other), natural conception (yes/no), folic acid intake before conception (yes/no), folic acid intake during pregnancy (yes/no), vitamins before

conception (yes/no), vitamins during pregnancy (yes/no), unprotected sex in the past 4 days (yes/no), parity (0-6), parity (0, >1), pre-existing conditions (yes/no), surgeries in the past 10 years (yes/no), smoking (yes/no), drinking alcohol (yes/no), antibiotics at enrolment (yes/no), gestational age at delivery (<37, ≥37), mode of delivery (vaginal, C-section), mode of delivery (vaginal, C-section, C-section-elective), fetal sex (male, female), neonate in level 2 care nursery (yes/no), birth weight (continuous variable) and Apgar score at 5 minutes (0-9).

Supplementary Table S1 (.xls)

Summary of OTU analyzed in this study. Best database match, percentage of identity, cpnDB name, taxonomic lineage and abundance in each library are shown.
